# 2024 global temperature record is consistent with model-predicted warming

**DOI:** 10.1073/pnas.2600021123

**Published:** 2026-05-11

**Authors:** Michael E. Mann, Byron A. Steinman, Alejandro Fernandez, Shannon A. Christiansen, Xueke Li

**Affiliations:** ^a^Department of Earth and Environmental Science, University of Pennsylvania, Philadelphia, PA 19104; ^b^Department of Earth and Environmental Sciences and Large Lakes Observatory, University of Minnesota Duluth, Duluth, MN 55812; ^c^Department of Earth and Environmental Sciences, University of Minnesota, Minneapolis MN 55405; ^d^Gulf Coast Repository, Texas A&M University, College Station, TX 77845

**Keywords:** climate change, temperature records, climate extremes

## Abstract

We employ a semiempirical approach combining climate model simulations and observational temperatures to assess the likelihood of recent global temperature records. Monte Carlo simulations are used to generate global temperature series consistent with combined estimates of forced (anthropogenic + natural) and internal variability derived from observations and CMIP6 multimodel simulations. We find that the El Niño-boosted 2024 global temperature record had a ~12% likelihood of occurrence (a one-in-eight-year event), similar to the prior (also El Niño-boosted) record year 2016 (~14% likelihood). Of the records set during the past three decades, only 1998 is found to have been truly anomalous, with a ~2.5% likelihood of occurrence. Each of these records is found to have been nearly impossible in the absence of human-caused warming.

## Significance

A number of recent articles and commentaries have argued that the record global temperatures of 2023–2024 cannot be explained by standard climate model simulations and infer that we are witnessing an unexpected surge in planetary warming. Using a semiempirical methodology that combines information from surface temperature observations and state-of-the-art multimodel climate simulations, we demonstrate that the recent global surface temperature spike is entirely consistent with expectations from the combined effects of model-predicted long-term warming and routine natural climate variability. Record warmth is found to have been extremely unlikely in the absence of human-caused warming.

The 2023/2024 El Niño event helped boost global mean surface temperature (GMST) in 2024 to record levels, beating the old (2016) record by a substantial margin (~0.25 °C). Some researchers have asserted, however, that the record warmth is inexplicable given understood climate drivers. One team (1), for example, published a New York Times op-ed “We Study Climate Change. We Can’t Explain What We’re Seeing.” Is it truly the case that we cannot explain the 2024 global temperature record? In other words, is the 2024 record consistent with standard climate model predictions?

In previous work based on earlier generation (CMIP5) multimodel simulations (2, 3) we showed that GMST records during the past two decades bear the clear fingerprint of human-caused warming. GMST for 1998, 2005, 2010, 2014, 2015, and 2016 were found to be virtually impossible (<2 × 10^−4^ % likelihood) in the absence of human-caused warming. By contrast, they were found to be unremarkable when human-caused warming is accounted for, with only 1998, boosted by the “El Niño of the century” (4), appearing somewhat anomalous relative to expectations in a warming climate.

A recent analysis by Terhaar et al (5) estimated that the 2023–2024 global sea surface temperature rise was a one-in-512-year event, terming the warming “unlikely but not unexpected.” Other recent analyses (6, 7), however, argue that 2023–2024 warming was not unlikely, explainable in terms of natural climate variability—i.e. the 2023/2024 El Niño event—superimposed on anthropogenic warming. In the current study, we attempt to resolve this ongoing debate, using a method designed specifically for assessing the likelihood of annual GMST records (2, 3).

Some past studies (8–10) have employed model-based fingerprint detection methods to study temperature extremes in a generic sense. In this approach, natural variability is estimated from the climate models themselves, which means that assessments of the likelihood of extremes are dependent on the models producing realistic natural variability—an assumption that is not necessarily justified. Another study (11) estimated the parameters of statistical noise models directly from the instrumental temperature record, without accounting for the impact of anthropogenic climate change on surface temperatures. Such a procedure likely yields artificially inflated estimates of the noise parameters (e.g., by overestimating the apparent degree of natural persistence and, hence, inflating estimated natural likelihoods of extreme events).

The Terhaar et al. (5) study instead used both a small set of model simulations and observational data but employed a detrending that removed all variations with timescales longer than 40 y prior to estimating noise parameters. The forced (signal) and internal (noise) components of variability cannot be properly distinguished simply by such a detrending, however, since each contribution contains components across the full range of timescales (12). Instead, an explicit model is needed for both signal and noise. Our approach (2, 3) addresses such considerations.

## Estimating the Likelihood of Record Global Temperatures

We represent GMST through a statistical model of the form

[1] T(t)=F(t)+I(t),

where *T*(*t*) is GMST*, F*(*t*) is the total forced response, *I*(*t*) represents the internal (noise) component of natural variability, and

[2] F(t)=A(t)+N(t),

where *A*(*t*) represents the forced anthropogenic (greenhouse gases, aerosols, and human land use) component of temperature change and *N*(*t*) represents the forced natural (volcanic + solar) component. *I*(*t*) is dominated by the El Niño/Southern Oscillation (ENSO) on interannual timescales, but there are other potential contributions on decadal and longer timescales due to coupled ocean-atmosphere variability in the Pacific and Atlantic oceans (13).

Observational data provide a real-world estimate of *T*(*t*) which we will call *T*_0_(*t*), while climate model simulations can be used to provide an estimate of *F*(*t*), which we will call *F*_0_(*t*). *F*_0_(*t*) is estimated by averaging across a large ensemble of simulations subject to common forcing such as the Coupled Model Intercomparison Project (CMIP) multimodel simulations (while we will henceforth refer to these experiments as multimodel ensembles, strictly speaking they are *pseudoensembles* since it is not just initial conditions, but model physics that varies among the individual simulations). The averaging process isolates the purely forced component of surface temperature change, since each simulation contains a single, unique realization of the underlying stochastic noise process, the sum of which cancels when averaged over a large number of independent realizations (2, 3, 14). The difference *T*_0_(*t*) *− F*_0_(*t*) can be interpreted as an estimate of the pure internal variability component (*I*) which we will call *I*_0_(*t*). It is diagnosed as the residual series after subtracting *F*_0_(*t*) from *T*_0_(*t*). However, *I*_0_(*t*) represents only one possible realization (the actual realization that we have historically experienced) of the presumed underlying noise process. It is appropriate to define a more general stochastic time series model for *I*(*t*) using parameters estimated from this one realization.

Here, we make use of the most recent (CMIP6) multimodel suite of climate model simulations (15) and observational surface temperature data to estimate *F*_0_(*t*) and *T*_0_(*t*), and we fit a statistical model to the residual series *I*_0_(*t*) = *T*_0_(*t*) *− F*_0_(*t*) to generate a large ensemble of internal variability sequences from which we can derive a statistical distribution of alternative global temperature histories, *T*_(*n*)_(*t*), that are consistent with both models and observations.

While further technical details are described in the *Materials and Methods* and *SI Appendix*, a few additional methodological considerations are worthy of note. As in previous work (14), we allow for a scaling *F*_0_(*t*) = *β F*_MMM_(*t*) where the factor *β* (which can differ from unity) relates our estimate of the forced temperature component *F*_0_(*t*) to the GMST multimodel mean series *F*_MMM_(*t*). *β* is determined via linear regression of the instrumental temperature series *T*_0_(*t*) against the multimodel mean series *F*_MMM_(*t*) during the time interval of overlap (We used the common period of model/observation overlap (1880–2022) which precedes the 2023/2024 warming event).

The rescaling procedure is warranted for two separate reasons. First, there is an incongruity between what is measured with standard observational GMST products (a blend of Surface Air Temperature or “SAT” over land and Sea Surface Temperature or “SST” over ocean) and what is typically reported for modeled global mean surface temperatures (SAT over all regions). Model GMST series therefore tend to overestimate warming relative to their observational counterpart since SAT increases more than SST over common oceanic regions in anthropogenic warming scenarios. Past work has attempted to deal with this problem by sampling the models through a similar blend of SAT over land and SST over water (see ref.s (2, 3)). However, even in this case, there is still a potential mismatch due to changing spatial coverage and time-evolving sea ice cover in the observations (16). One approach to this problem recognizes that there is an undetermined scaling factor between observational and model surface temperature series which can be empirically estimated as above, recognizing caveats regarding the use of a constant scaling factor in the presence of time-dependent spatial sampling.

Second, it would be fortuitous if the true sensitivity of the climate system were precisely equal to the multimodel ensemble mean. There is notable disagreement even between multimodel mean equilibrium climate sensitivity (ECS) estimates from the two most recent multimodel intercomparison projects, with CMIP5 yielding an average of ~3.3 °C (range 2.1 to 4.7 °C) and CMIP6 yielding an average of ~3.8 °C (range 1.8 to 5.6 °C) (17). Some researchers (18) have argued for screening the CMIP6 multimodel ensemble to remove so-called “hot models” deemed to have unphysically high transient climate responses (this yields an average ECS of ~3.4 °C instead). But this process could be criticized for subjectivity (for example, one might contest the metrics and criteria by which particular models have been removed), and it could in principle lead to a bias. The allowance of an empirically determined scaling factor *β*, as defined above, alleviates the need for any ad hoc screening.

The internal variability or “noise” component *I*(*t*), as in earlier work (2, 3), is modeled using a general linear stationary time series model of the form ARMA(*p*,*q*), where *p* and *q* are the order of the autoregressive and moving average components, respectively. The optimal values of these parameters (*Materials and Methods*) can be chosen alternatively based on either the Akaike Information Criterion (AIC) or Bayesian Information Criterion (BIC), each of which weigh model fit against parsimony differently. From a relative standpoint, BIC tends to give more weight to the latter while AIC gives more weight to the former. While we feature AIC in our standard analyses, we perform parallel analyses using BIC (*SI Appendix*).

The statistical model is fit to *I*_0_(*t*) over the common period of overlap 1880–2022 preceding the 2023/2024 warming event. Monte Carlo simulations are then used to produce an ensemble of surrogate noise series *I*_(n)_(*t*) which are added to *F*_0_(*t*) to yield surrogate temperature series *T*_(n)_(*t*) that extend through 2024. To assess sensitivity to the specific observational data used, we perform parallel analyses using three alternative GMST datasets including the HadCRUT5 (19), GISTEMP v4 (20), and Berkeley Earth (21) GMST datasets (*Materials and Methods*).

Our standard analysis is based on the *N_expanded_* = 44 simulations ensemble obtained by combining the *N_standard_* = 33 CMIP6 models available from the official Lawrence Livermore National Laboratory (LLNL) CMIP6 multimodel archive with 11 additional simulations archived by the E.U. Copernicus site (*SI Appendix*). We perform a parallel set of analyses using only the *N_standard_* = 33 models. In each case, we perform analyses using both the full multimodel ensembles and alternative “screened” versions of those ensembles wherein putative “hot models,” as diagnosed by transient climate response or “TCR” (18) have been selectively removed (*SI Appendix*, Table S1).

Our analysis is confined to the historical time interval 1880–2024, during which complete years for all three observational datasets and CMIP6 simulations are available. The historical simulations from 1880–2014 were extended through 2024 using projections from the corresponding models that follow the SSP2-4.5 emissions scenario, argued to constitute the closest match to actual emissions during this intervening time period (22). GMST in the models was defined by the global spatial mean of the model SAT field over both land and ocean regions (*Materials and Methods*).

For each experiment, we performed *N* = 40,000 Monte Carlo simulations to produce an ensemble of *N* surrogate internal variability series *I_(n)_*using the selected AR(*p*,*q*) noise model (*Materials and Methods*). For all six experiments, an ARMA(1,2) noise model was selected using the standard (AIC) selection criterion. For the alternative experiments using BIC in place of AIC, an AR(1), i.e., simple “red noise” model, was selected in each case. The autocorrelation functions of the ARMA innovation sequences are found to display little structure, supporting the overall adequacy of the fitted ARMA noise models in capturing the basic character of the residual series. The estimated red noise decorrelation times τ for *I*_0_(*t*) are between roughly 1.5 and 2 y (*Materials and Methods*), implying that the noise fluctuations for neighboring years are highly correlated. For this reason, record or near-record temperatures often occur for multiple successive years (e.g., for 2014–2016 and 2023–2025).

The noise realizations were added to the estimated forced temperature series *F*_0_(*t*) to yield an ensemble of *N* surrogate GMST series *T*_(n)_(*t*). We then calculated the fraction of the *N* surrogates for which the actual recorded GMST value *T*_0_(*t*) was exceeded for each year of the past three decades during which a new record was established at the time: 1998, 2005, 2010, 2014, 2015, 2016, 2023, and 2024.

We performed a parallel set of analyses to assess the likelihood of breaching these observed record temperature thresholds from natural variability alone. These analyses were alternatively performed using both the standard AIC-selected (main article) and alternative BIC-selected (*SI Appendix*) ARMA(*p*,*q*) noise model and additionally, for a “persistent red noise” model. In this latter case, the lag-one autocorrelation ρ is evaluated using the raw observational series *T*_0_(*t*) rather than the estimated residual series *I*_0_(*t*). Such a noise model could be considered unphysical, since the inferred noise amplitude and persistence are both inflated substantially by the presence of the (nonstationary) anthropogenic warming trend (the corresponding noise decorrelation time is τ ~ 17 y; see *Materials and Methods*). It is thus used as an overly liberal, extreme upper bound estimate on the chance occurrence of temperature extremes in the absence of anthropogenic warming.

## Comparison of Modeled and Observed Global Temperatures

**Fig. 1.**
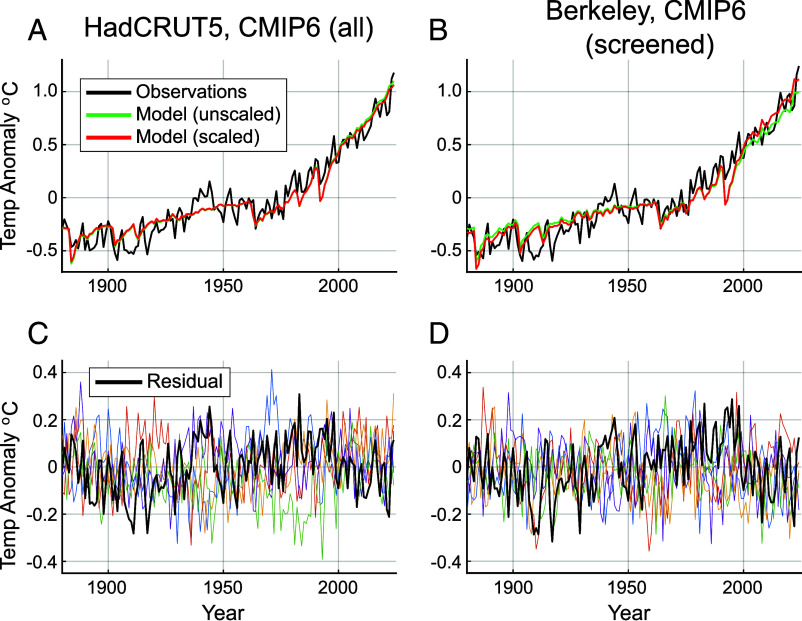
Modeled vs. Observed GMST (1880–2024) for standard case (expanded CMIP6 model set and AIC selection criterion). *Top*: Comparison of unscaled [*F*_MMM_(*t*)] and scaled [*F*_0_(*t*)] CMIP6 multimodel means with observations using the (*Left*) CMIP6 all/HadCRUT (*A*) and (*Right*) CMIP6 screened/Berkeley (*B*) model/observation combinations. *Bottom*: Corresponding (*C*, *D*) residual (observations minus scaled model) series [*I*_0_(*t*)] (thick black curve) along with 20 representative Monte Carlo surrogates [*I*_(*n*)_(*t*)] (colored curves). Here, and in subsequent figures, anomalies are relative to the mean over the full period 1880–2024.

We show results based on six different experiments, alternatively employing the three different observational GMST temperature products and both CMIP6 “all” and CMIP6 “screened” model GMST series, for our standard (AIC selection criterion and expanded CMIP6 model set) case (Table 1; see *SI Appendix*, Table S2–S4 for results using BIC and restricted CMIP6 set). Fig. 1 compares the multimodel mean-estimated forced temperature histories against the observed temperature histories *T*_0_(*t*) for two of these experiments: 1) HadCRUT5 for *T*_0_(*t*) and CMIP6 “all” for *F*_0_(*t*) (experiment #1 in Table 1) and 2) Berkeley Earth for *T*_0_(*t*) and CMIP6 “screened” for *F*_0_(*t*) (experiment #6 in Table 1). Results for the four other experiments are qualitatively similar (*SI Appendix*, Fig. S5–S8). The use of BIC rather than AIC in statistical modeling also yields qualitatively similar results (*SI Appendix*, Tables S2-S4 and Fig. S9-S11). The use of the restricted LLNL-only CMIP6 multimodel ensemble also yields similar results (*SI Appendix*, Tables S2–S4).

**Table 1.** Details of experiments (CMIP6 standard/AIC) with estimated likelihoods (in %) for standard case (expanded CMIP6 model set and AIC selection criterion)^*^

Experiment #	ARMA(p,q)	β	1998	2005	2010	2014	2015	2016	2023	2024
1. HadCRUT /CMIP6 All	ARMA(1,2)	0.97								
Anthropogenic + Natural			2.1	33	43	69	32	15	22	15
Natural (ARMA)			0	0	0	0	0	0	0	0
Natural (Persist Red Noise)			6.5	5.5	3.7	3.8	1.5	0.71	0.14	0.07
2. HadCRUT /CMIP6 Screen	ARMA(1,2)	1.05								
Anthropogenic + Natural			1.5	30	29	69	23	12	20	11
Natural (ARMA)			0	0	0	0	0	0	0	0
Natural (Persist Red Noise)			6.5	5.5	3.7	3.8	1.5	0.71	0.14	0.07
3. GISTEMP /CMIP6 All	ARMA(1,2)	0.94								
Anthropogenic + Natural			4.1	29	48	62	26	10	20	6.0
Natural (ARMA)			0	0	0	0	0	0	0	0
Natural (Persist Red Noise)			7.4	4.9	4.0	3.3	1.2	0.45	0.17	0.05
4. GISTEMP /CMIP6 Screen	ARMA(1,2)	1.02								
Anthropogenic + Natural			3.3	26	34	62	18	8.4	18	4.2
Natural (ARMA)			0	0	0	0	0	0	0	0
Natural (Persist Red Noise)			7.4	4.9	4.0	3.3	1.2	0.45	0.17	0.05
5. Berkeley /CMIP6 All	ARMA(1,2)	1.04								
Anthropogenic + Natural			2.3	31	51	74	42	17	32	19
Natural (ARMA)			0	0	0	0	0	0	0	0
Natural (Persist Red Noise)			6.4	5.3	4.3	4.2	1.8	0.75	0.24	0.11
6. Berkeley /CMIP6 Screen	ARMA(1,2)	1.12								
Anthropogenic + Natural			1.5	28	37	75	32	14	30	15
Natural (ARMA)			0	0	0	0	0	0	0	0
Natural (Persist Red Noise)			6.4	5.3	4.3	4.2	1.8	0.75	0.24	0.11

^*^“0” = No occurrences in 40,000 simulations.

We show (Fig. 1 *A* and *B*) both the unscaled [*F*_MMM_(*t*)] and scaled [*F*_0_(*t*)] model series. For the first case (experiment #1), the scaling parameter *β* is slightly below unity, meaning that the best fit of the multimodel mean series to the instrumental series is afforded by reducing its amplitude slightly. For the second case (experiment #6), we encounter the opposite situation. The individual values range from *β* = 0.94 to *β* = 1.12 among the six experiments, with values below unity on average for experiments using the full CMIP6 ensemble (consistent with modeled warming that is artificially amplified relative to observed warming) and values above unity on average for experiments using the screened CMIP6 ensemble (consistent with modeled warming that is artificially diminished relative to observed warming). Interestingly, the average value of the scaling parameter *β* over the six different experiments (Table 1) is remarkably close to unity (*β* = 1.02). That seems fortuitous given the absence of any a priori reason to expect that the two competing effects discussed above (data/model surface temperature field incongruity and potential mismatch between real world and modeled climate sensitivity) should almost precisely cancel on average.

The most notable discrepancy between models and observations in recent decades (Fig. 1 *A* and *B*) corresponds to the 1991 Mt. Pinatubo eruption in the Philippines. The models predict substantially greater posteruption cooling than the observations. The eruption was coincident with an emerging El Niño episode in the early 1990s that likely offset a substantial fraction of the predicted postvolcanic cooling. A number of studies have argued that tropical volcanic forcing may in fact predispose the climate toward El Niño (23, 24), a response that is not generally captured in climate models (25). The real world validity of this mechanism continues to be debated in the literature (25–27) as does the possibility that anthropogenic forcing itself may interact with internal ENSO-related variability (28). One could argue that this and other such model/data mismatches may reflect a structural error in the model response itself rather than a manifestation of anomalous internal variability.

Larger departures are evident, in general, earlier in the record (particularly using HadCRUT5). Of special note is the period 1942–1945 when observational sea surface temperatures may exhibit spurious warmth due to a bias in bucket and direct intake ship-based SST measurements that is exaggerated by reduced sampling during WW II (29). Similar model/observation discrepancies have been noted for the period 1900–1920 (30–32). Such discrepancies could lead to a modest artificial inflation of the residual variance and, in turn, amplified simulated random departures and a greater, rather than lesser, likelihood of breaching particular recent warming thresholds.

These same features are readily observed in the residual series *I*_0_(*t*) which is shown along with its Monte Carlo surrogates *I*_(*n*)_(*t*) in Fig. 1 *C* and *D*. We also observe notable positive peaks during El Niño years such as 1982/83, 1991/92, and the unusually extended early 1990s El Niño (33). The persistent negative values from the late 2000s through the early 2010s and again during the late 2010s and early 2020s are associated with unusually persistent “multiyear La Niñas” that have become increasingly common in recent decades, possibly itself a consequence of anthropogenic warming (34). In comparison with these features, the El Niño years 2016 and 2024 appear as relatively unremarkable positive peaks in the residual series. The surrogates *I*_(*n*)_(*t*) capture the general character of the actual residual series *I*_0_(*t*) quite well, though certain prominent features in *I*_0_(*t*), i.e. the 1900–1920 negative peak and WWII positive peak, both of which may be associated with observational biases, are especially prominent in *I*_0_(*t*).

## How Likely Was the 2024 Global Temperature Record?

**Fig. 2.**
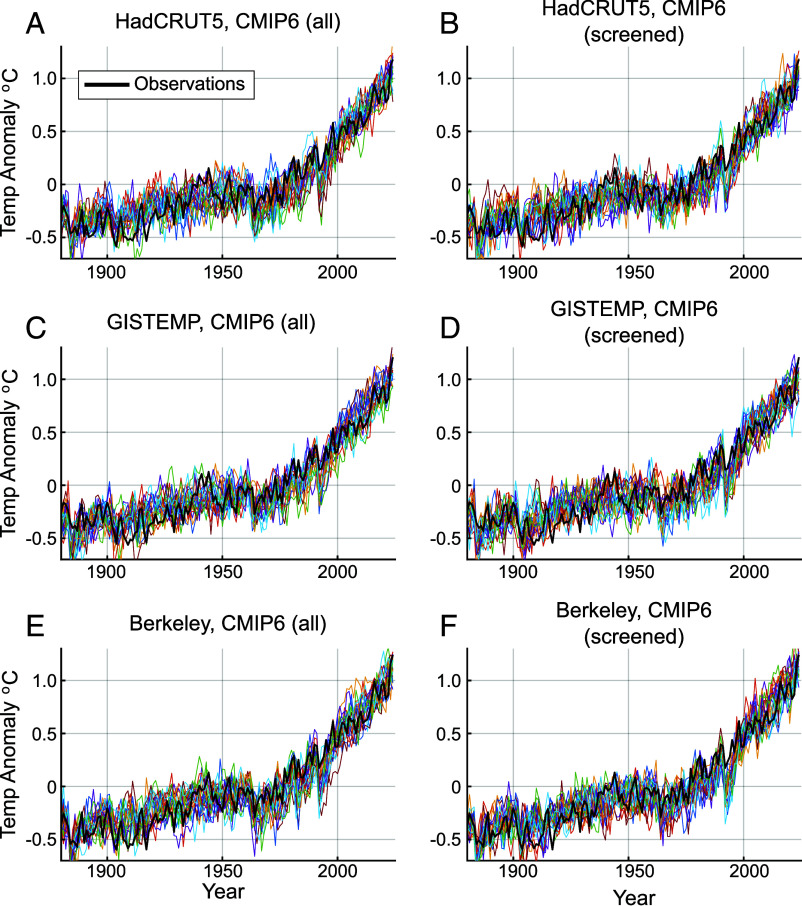
Observed GMST vs. Monte Carlo GMST Surrogates (1880–2024) for each of the six experiments described in the article (standard case). (*A*) HadCRUT5, CMIP6 (all), (*B*) HadCRUT5, CMIP6 (screened), (*C*) GISTEMP, CMIP6 (all), (*D*) GISTEMP, CMIP6 (screened), (*E*) Berkeley, CMIP6 (all), and (*F*) Berkeley, CMIP6 (screened) as described in Table 1. The observations (thick black curve) are compared in each case with 20 representative Monte Carlo GMST surrogates [*T*_(*n*)_(*t*)] (colored curves).

In Fig. 2, we show a representative sample of global temperature surrogates *T*_(*n*)_(*t*) that reflect a combination of anthropogenic and natural climate variability, along with the actual instrumental surface temperature history *T*_0_(*t*), for each of the six experiments based on the BIC criterion (see *SI Appendix* for results based on AIC). The *T*_0_(*t*) values in general lie well within the distribution of surrogates *T_(n)_* for all six cases. One clear exception is the years immediately following the 1991 Pinatubo eruption where the observations lie at the very upper (warm) end of the distribution of surrogates, for reasons discussed earlier. Another exception is 1998, associated with the “El Niño of the century,” wherein observed global temperatures are seen to rise above the distribution of surrogates. The El Niño-boosted 2024 global temperature, by contrast, is seen to lie within the distribution of surrogates. The likelihoods of reaching or exceeding the levels reached for specific years, as calculated from the full sample of *N*=40,000 Monte Carlo surrogates for all six experiments, are tabulated in Table 1 (see *SI Appendix* for AIC-based results and for results based on restricted LLNL-only CMIP6 models). The 1998 global temperature is exceeded only 1.5 to 4.1% (average of 2.5%) of the time, a relatively rare, roughly one-in-forty-year event. By contrast, the 2024 global temperature is exceeded 4.2 to 19% (average of 12%) of the time, a far less anomalous (roughly one-in-eight-year) event that is likely to have happened at least once since 2017. We obtain qualitatively similar results in the alternative experiments (i.e., using BIC or the restricted LLNL model set; see *SI Appendix*), with the 1998 global temperature exceeded 1.5 to 5.5% (average of 3%) of the time, and the 2024 global temperature exceeded 4.2 to 20% (average of 11%) of the time.

**Fig. 3.**
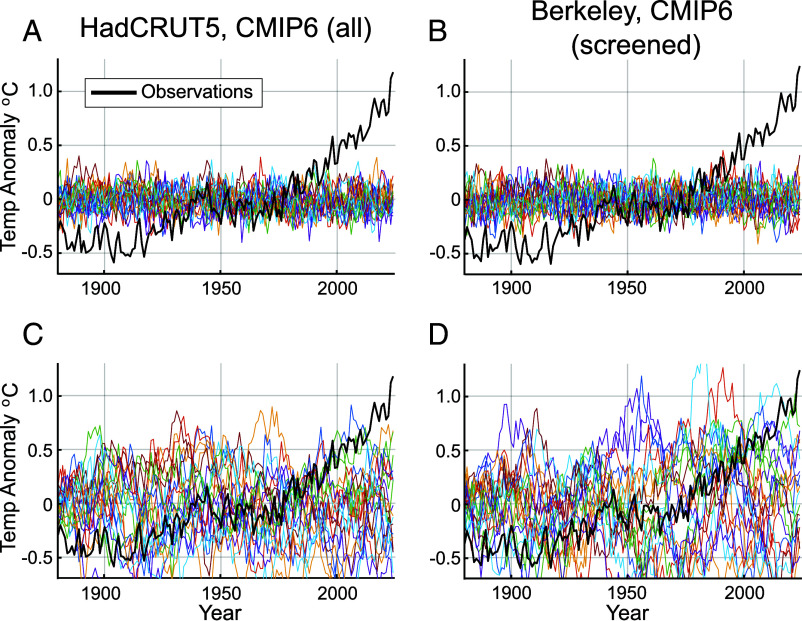
Observed GMST vs. Monte Carlo GMST Surrogates (1880–2024) without anthropogenic component for standard case for both ARMA noise (*Top, A, B*) and persistent red noise (*Bottom, C, D*). Shown are results for (*Left, A, C*) CMIP6 all/HadCRUT and (*Right, B, D*) CMIP6 screened/Berkeley experiments. As in Fig. 2, 20 representative Monte Carlo GMST surrogates [*T*_(*n*)_(*t*)] (colored curves) are shown.

It is finally also of interest to examine the case where only natural variability is accounted for. In Fig. 3, we show representative samples of global temperature surrogates *T*_(*n*)_(*t*) for two experiments (the other four experiments are shown in *SI Appendix*) where natural variability alone is represented. We alternatively use ARMA noise (Fig. 3 *A* and *B*) and “persistent red noise” (Fig. 3 *C* and *D*). In the former case, the surrogates never come close to breaching any of the record temperatures of the past three decades, with not a single surrogate out of 40,000 exceeding these thresholds (Table 1). In the latter case, with its artificially large long-term natural fluctuations, these records are occasionally breached, but successively warmer, more recent records are increasingly rare, with the 2024 temperature exceeded only 0.05 to 0.11% of the time, i.e., a less than roughly once-in a millennium (~900 to 2,000) year event (Table 1). It is fair to say that the 2024 global temperature record was exceptionally unlikely in the absence of human-caused warming, even using this arguably unphysically liberal noise model.

### Conclusions.

We find the El Niño-boosted 2024 global temperature record to have been *unremarkable*—specifically, a roughly one-in-eight-year event—when anthropogenic forcing is accounted for. Among all record-breaking years during the past three decades, only 1998, boosted by what has been termed the “El Niño of the century,” as a roughly one in forty-year event, was found to be anomalous. These conclusions are robust to screening “hot models” (as defined in ref. 18) from the CMIP6 ensemble. By contrast, we find the 2024 record (and other recent records) to have been exceptionally *unlikely* in the absence of human-caused warming.

One objection that might be raised is that the historical CMIP6 simulations we have employed are based on projections after 2015 and do not account for recent changes in radiative forcing unanticipated a decade ago. An example is the January 2022 Hunga Tonga–Hunga Ha’apai eruption in the south Pacific. Recent studies, however, suggest that the radiative forcing from this eruption was negligible by the end of 2023 (35). Other studies argue for an increase in positive radiative forcing over the tropical Atlantic since 2020 due to changes in policies regarding ship-borne sulfur emissions (36). However, the most comprehensive assessments estimate at most a roughly +0.09 Wm^−2^ increase in global mean radiative forcing from 2020 to 2024 in association with this change (37), which translates to a very small (<0.03 °C) warming effect by 2024 (38). Furthermore, it is worth noting our use of SSP2-4.5 projections (following ref. 22) to extend simulations from 2015–2024 reflects an arguably conservative assumption. If higher emissions scenarios are a better characterization of recent historical emissions as argued by some researchers (39), then our analysis will modestly underestimate, rather than overestimate, the likelihood of recent temperature extremes.

Our findings, finally, are relevant to the recent debate about whether the 2023–2024 record global warmth is indicative of an unexpected surge in global warming (e.g., ref. 36). Consistent with the conclusions of Beaulieu et al., (40), we find no evidence for any departure from the expectations of typical natural climate variability superimposed on model-predicted anthropogenic warming. Recent record warmth, in conclusion, does not contradict state-of-the-art climate model historical simulations and future projections of GMST. Such models continue to provide accurate assessments of planetary warming and a sound basis for climate policymaking.

## Materials and Methods

### Observational Surface Temperature Data.

We used annual mean global combined land/ocean temperature series as computed by three leading groups including 1) HadCRUT5 (20), available at http://www.metoffice.gov.uk/hadobs/hadcrut5, 2) GISTEMP v4 (21) available at https://data.giss.nasa.gov/gistemp/, and 3) Berkeley Earth (22) available at https://berkeleyearth.org/data/

### CMIP6 Simulations.

We used annual global temperatures series from the Coupled Model Intercomparison Project Phase 6 (CMIP6) (15) multimodel pseudoensemble, combining, as in Hausfather (41), the historical simulations from 1850–2014 with future (SSP2-4.5) projections from 2015–2030 to generate series from 1850–2024. Where multiple realizations for a given run were available, the first realization was used as per Hausfather (41). The requirement of continuous common data over both time periods applied to the official CMIP6 Lawrence Livermore National Laboratory (LLNL) (https://aims2.llnl.gov/search) archive yielded 33 total models. Additional CMIP6 model simulations archived at the E.U. Copernicus site (https://cds.climate.copernicus.eu/datasets/projections-cmip6) yielded an expanded set of 44 total models satisfying these criteria.

We used the larger *N_expanded_* = 44 set of model simulations in the main article but similar results were obtained using the smaller *N_restricted_* = 33 set (*SI Appendix*).

Using the TCR criterion of Hausfather et al (41), we identified smaller “screened” subsets of models in which so-called “hot models” were removed, yielding *N_screened(restricted)_*=19 for the restricted LLNL set and *N*_*screened*(*expanded*)_ = 23 for the expanded LLNL+Copernicus set.

It should be noted that the residual internal variability that survives after averaging over the multimodel ensemble is not zero. It scales as 1/*N^1/2^*, where *N* is the sample size. For example, for our full set of *N_expanded_* = 44 simulations, if the typical amplitude of internal variability in a single realization is 0.1 °C, then we expect the multimodel mean to have a residual nonzero internal variability component of ~0.015 °C, which is quite small compared to the amplitude of the components of our statistical model. For smaller multimodel ensembles (e.g., the “hot model” subset), the residual component is modestly greater.

### Details of Statistical Modeling Exercises.

The ARMA(*p*,*q*) model for the residual series contains *p* autoregressive terms (the “AR” part of the model) and *q* moving-average terms (the “MA” part of the model), taking the form

[S1] $$y_{t}=c+[a_{1}y_{t-1}+\cdots +a_{p}\varepsilon_{t-p}]+[b_{1}\varepsilon_{t-1}+\cdots +b_{q}\varepsilon_{t-q}]+\varepsilon_{t},$$

where the “innovation” sequence *ε_t_* is assumed to conform to Gaussian white noise. Since the long-term mean is removed for all-time series in our analysis over the common time period analyzed, *c* is zero in Eq. S1 in our simulations. The AR(1) “red noise” model is a special simplified case where only *a*1 is nonzero in Eq. **S1**.

The selection of *p* and *q* in the ARMA(*p*,*q*) time series model was accomplished for each series by alternatively minimizing the Akaiki Information Criterion (AIC) and Bayesian Information Criterion (BIC), which is calculated based on the log likelihood function and number of parameters *n* = *p* + *q* + 1 (and in the case of BIC, an additional term *ln n*) for each fitted model. We confined the search radius to maximum values of *p* = *q* = 2, as larger values are arguably unphysical and/or lead to instability in the Yule–Walker equation solutions.

We show the autocorrelation function as a function of lag *l* = 1,…,20 for the estimated innovation sequences *ε_t_* diagnosed for each of the six experiments described in the main text, along with associated two-sided 95% confidence limits for Gaussian data for both AIC and BIC. Under the assumption of Gaussian white noise, we would expect an average of no more than 1 exceedance of the 95% bounds per experiment (i.e. 6 peaks summed over the six experiments). Using AIC (which tends to favor model fit over parsimony), we observe 1 or 2 peaks that reach or exceed the 95% level summed over the six experiments for the two CMIP6 sets (*SI Appendix*, Figs. S1 and S2). For BIC (which tends to favor parsimony over model fit), we observe (*SI Appendix*, Figs. S3 and S4) 13 peaks for the expanded CMIP6 set and 9 peaks for the restricted CMIP6 set. Under Poisson statistics, the sampling uncertainty for a count of *N* is ±*N^1/2^* (e.g., ±3 for *N* = 9). We thus conclude that there is no compelling evidence of nonrandom structure in the innovation series for 3 of the 4 cases, but additional caveats are appropriate for the one case (BIC/expanded CMIP6 set) where there is modest evidence for residual unresolved structure.

For the ARMA-based natural-only experiments (Fig. 3 *A* and *B* in the main article), by contrast with our earlier studies (2, 3), we did not include a forced (solar + volcanic) natural component. This component is not available for the CMIP6 multimodel simulations per se (estimating this component would involve subtracting the anthropogenic-only CMIP6 run means from the multimodel all-forcing CMIP6 run means; since the two respective multimodel pseudoensembles do not include an identical set of models, combining the multimodel means in this way would impart a potential bias). The solar-forced component is extremely small and has no meaningful impact on the estimated occurrence of warm extremes. Since volcanic forcing is one-sided (i.e. it only leads to global mean cooling), our neglect of this contribution implies that our Monte Carlo surrogates in these particular experiments are biased on the warm side in the years immediately following large volcanic forcing events. That means that the true likelihood of naturally exceeding observed temperatures during these years is likely lower than our estimates, making our procedure, if anything, overly conservative with respect to rejection of the null hypothesis that observed temperatures can be explained by natural variability. It should also be noted that our approach here makes an implicit assumption that the long-term (1880–2022) mean represents a natural baseline. To the extent that this mean is already elevated by anthropogenic warming, our estimates of exceedance likelihood estimates are once again conservative.

For the “persistent red noise” natural-only ensemble (Fig. 3 *C* and *D* in the main article), we fit a simple AR(1) model to the raw instrumental temperature series, setting *a*1 of Eq. S1 equal to ρ where ρ is the lag one autocorrelation coefficient for each of the instrumental series over the 1880–2022 interval used to define our statistical model (ρ = 0.94 for each of the three instrumental temperature datasets HadCRUT5, GISTEMP, and Berkeley Earth). This is associated with a decorrelation time of τ = 1/ln ρ ~ 17 y. By contrast, the autocorrelation times for the residual series *I*_0_(*t*) range from ρ = 0.49 to ρ = 0.57, corresponding to decorrelation times τ between 1.4 and 1.8 y.

## Supplementary Material

Appendix 01 (PDF)

## Data Availability

All study data are included in the article and/or *SI Appendix* and can also be found at https://doi.org/10.5281/zenodo.19556407 (42). Previously published data from the official CMIP6 Lawrence Livermore National Laboratory (LLNL; https://aims2.llnl.gov/search; (43)) and CMIP6 data archived at E.U Copernicus (https://cds.climate.copernicus.eu/datasets/projections-cmip6; (44) were used for this work.
