# Supplementary material for: 2024 global temperature record is consistent with model-predicted warming
Source: Proc Natl Acad Sci U S A. 2026 May 11;123(20):e2600021123. doi: 10.1073/pnas.2600021123 (PMC13187772; doi:10.1073/pnas.2600021123)
Supplement: Supplementary file 1 — Appendix 01 (PDF) [file pnas.2600021123.sapp.pdf]

Supplementary Materials for

2024 Global Temperature Record Is Consistent with Model-Predicted Warming

Michael E. Mann[1]*, Byron A. Steinman[2], Alejandro Fernandez[3], Shannon A. Christiansen[1], Xueke Li[1]

*Corresponding Author. Email: mmann00@upenn.edu

This PDF file includes:

Figs. S1 to S11
Tables S1 to S4

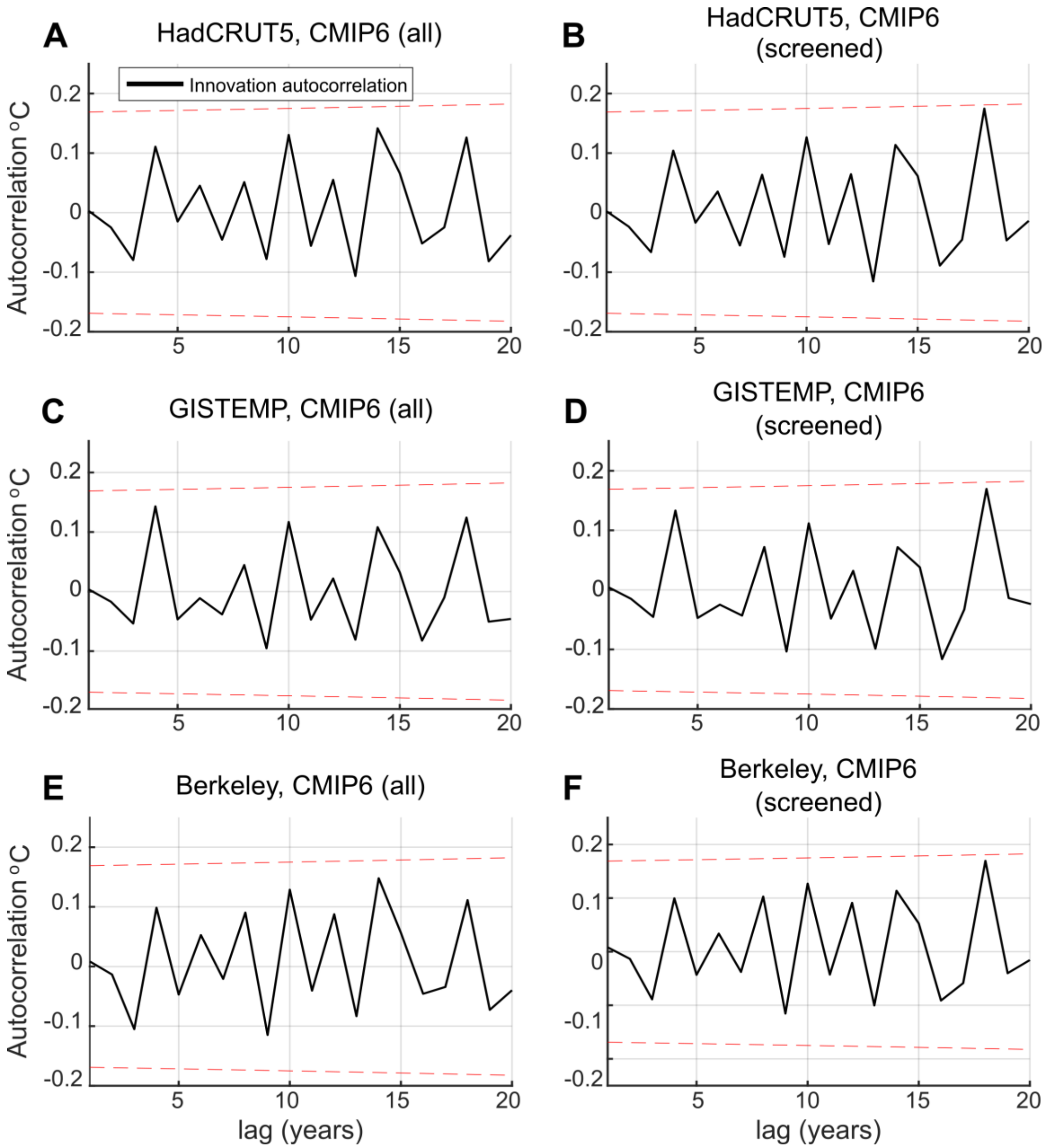

Fig. S1.

Autocorrelation (solid black curve) of estimated innovation series ε_t for each of the six experiments as a function of lag, along with two-sided 95% confidence limits (dashed black curves), standard (expanded) CMIP6 model set, standard (AIC) selection criteria.

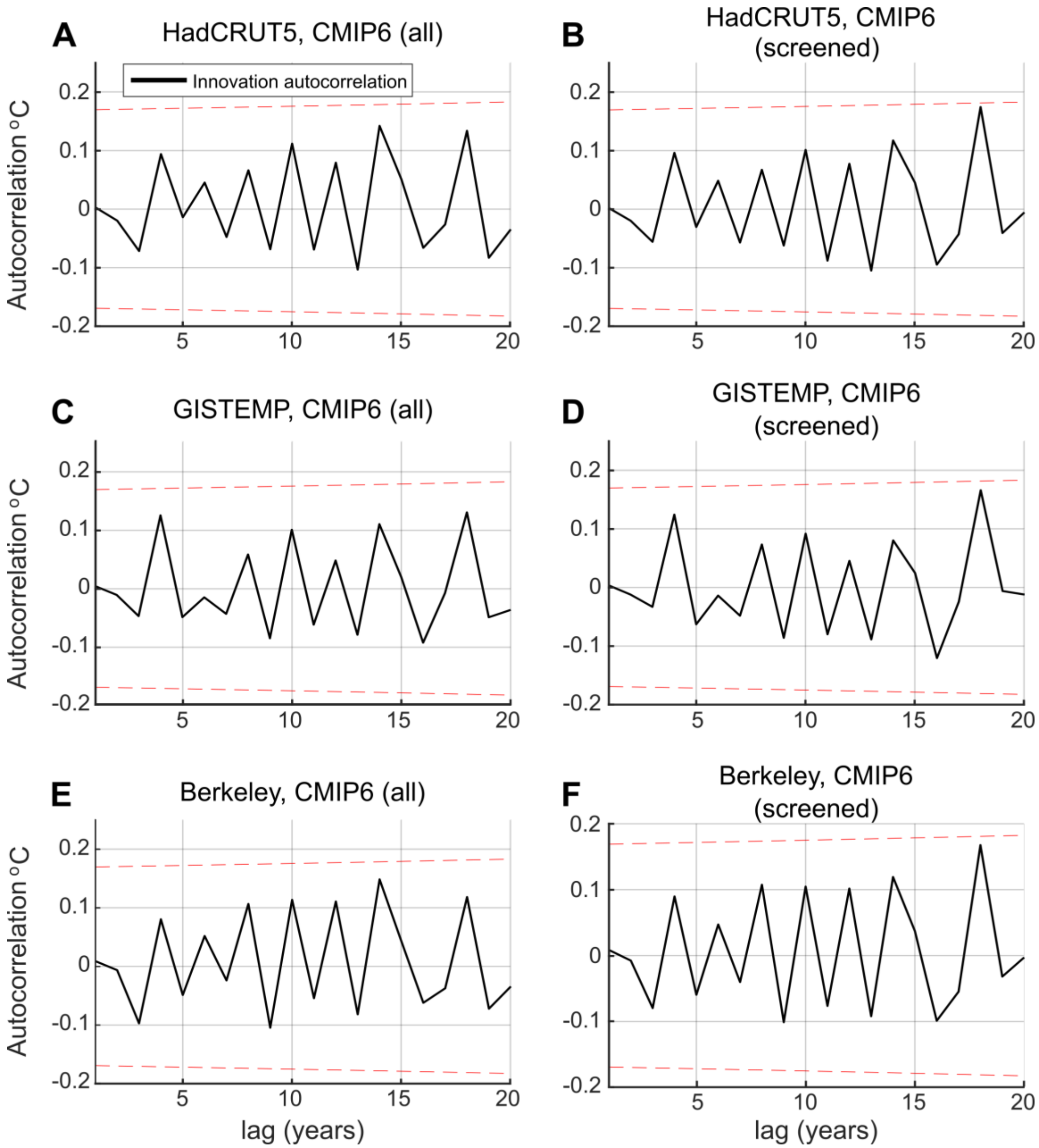

Fig. S2.

Autocorrelation (solid black curve) of estimated innovation series ε_t for each of the six experiments as a function of lag, along with two-sided 95% confidence limits (dashed black curves), alternative (restricted) CMIP6 model set, standard (AIC) selection criteria.

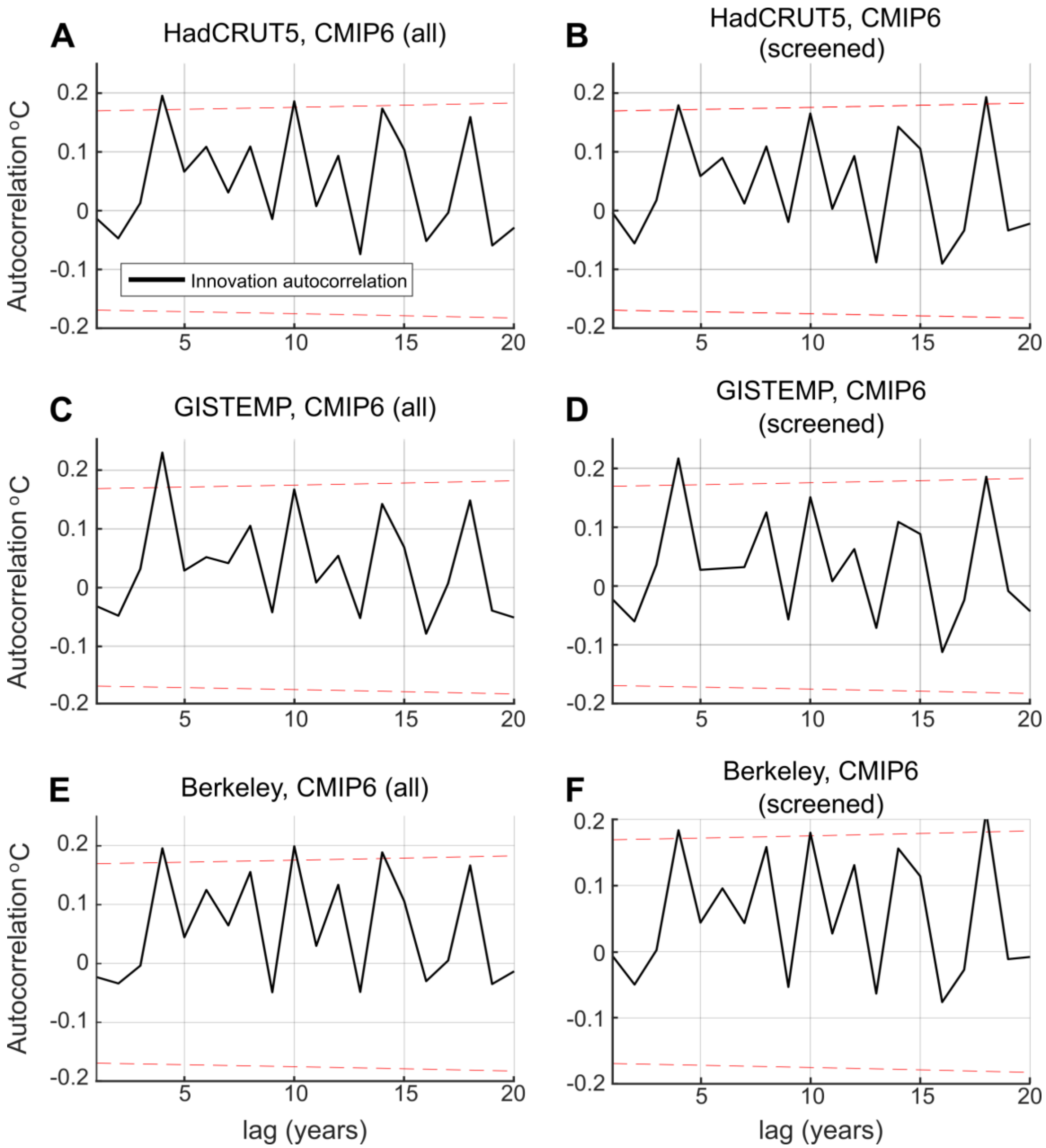

Fig. S3.

Autocorrelation (solid black curve) of estimated innovation series ε_t for each of the six experiments as a function of lag, along with two-sided 95% confidence limits (dashed black curves), standard (expanded) CMIP6 model set, alternative (BIC) selection criteria.

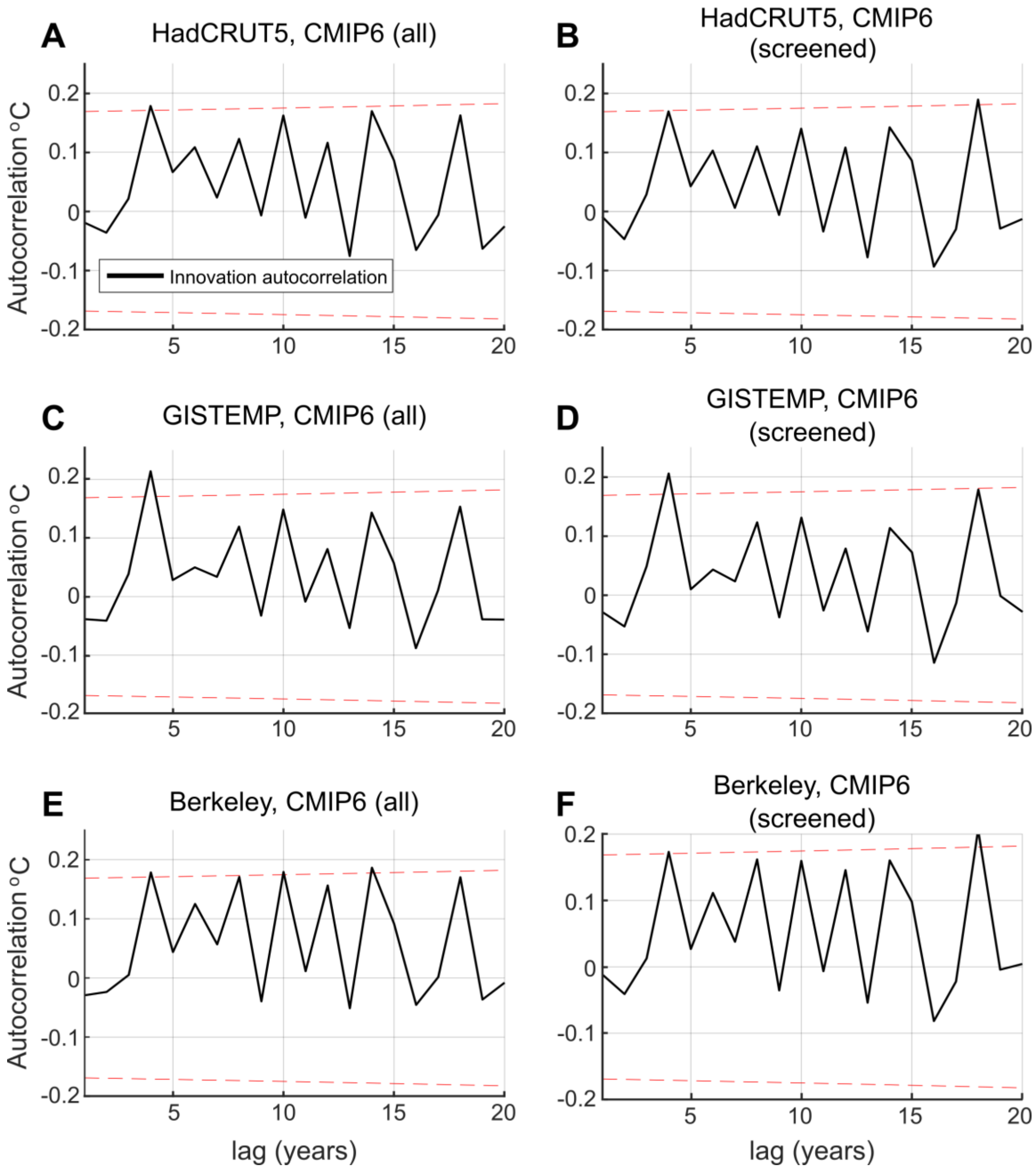

Fig. S4.

Autocorrelation (solid black curve) of estimated innovation series ε_t for each of the six experiments as a function of lag, along with two-sided 95% confidence limits (dashed black curves), alternative (restricted) CMIP6 dataset, alternative (BIC) selection criteria.

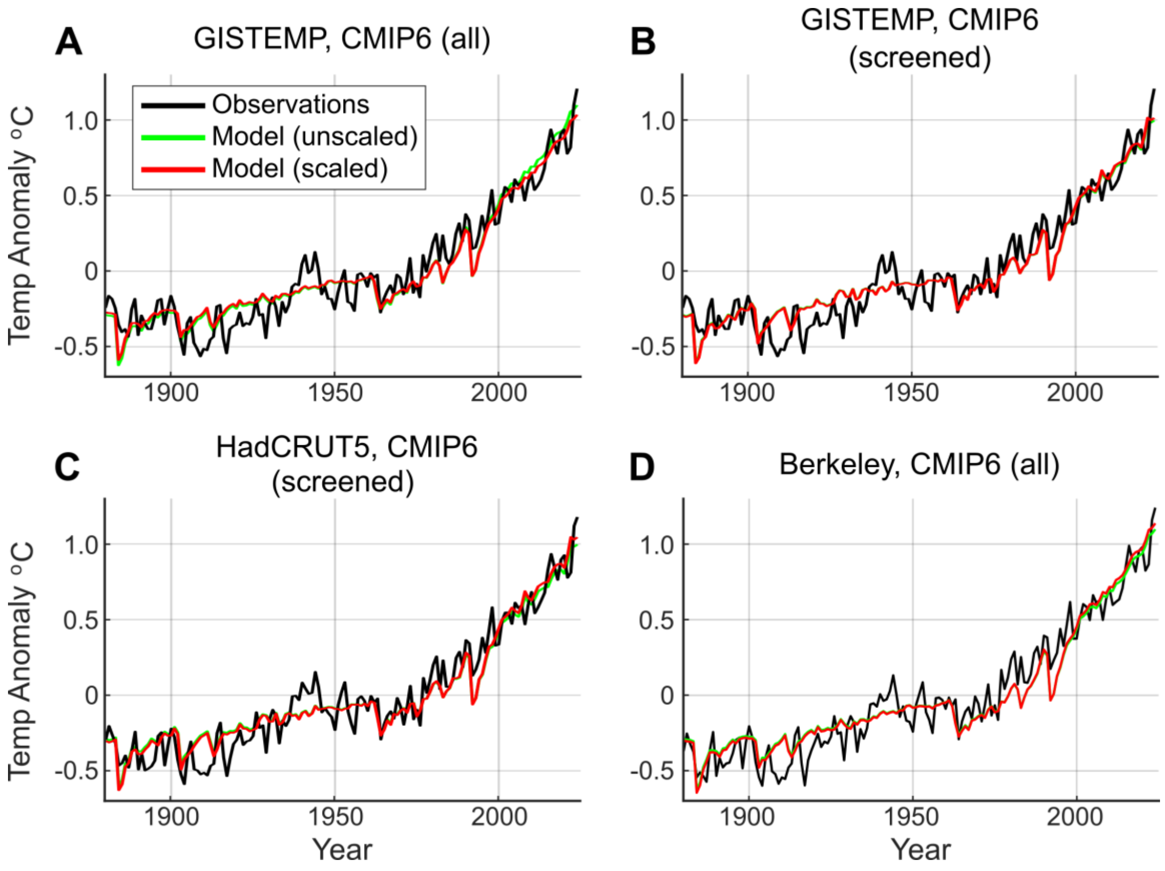

Fig. S5

As in Figure 1ab of main article, showing results for the other 4 experiments.

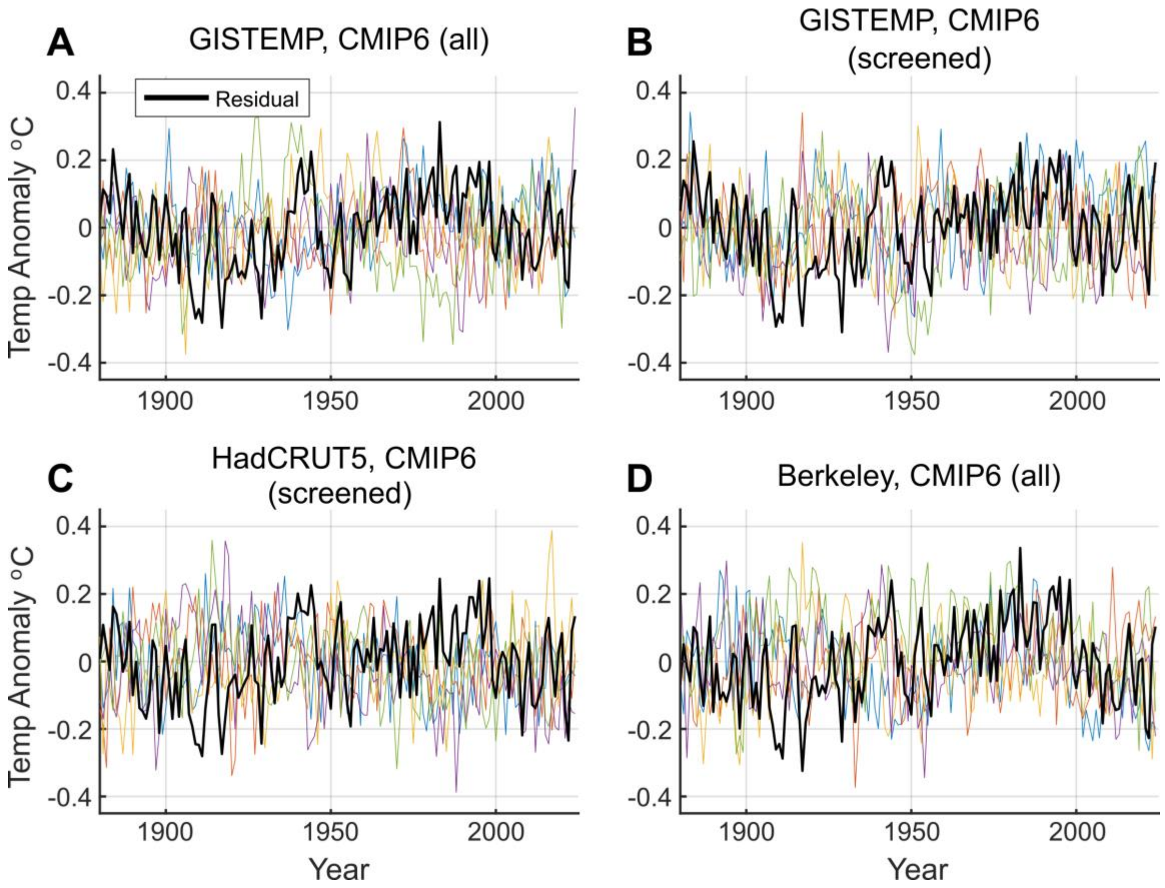

Fig. S6

As in Figure 1cd of main article, showing results for the other 4 experiments.

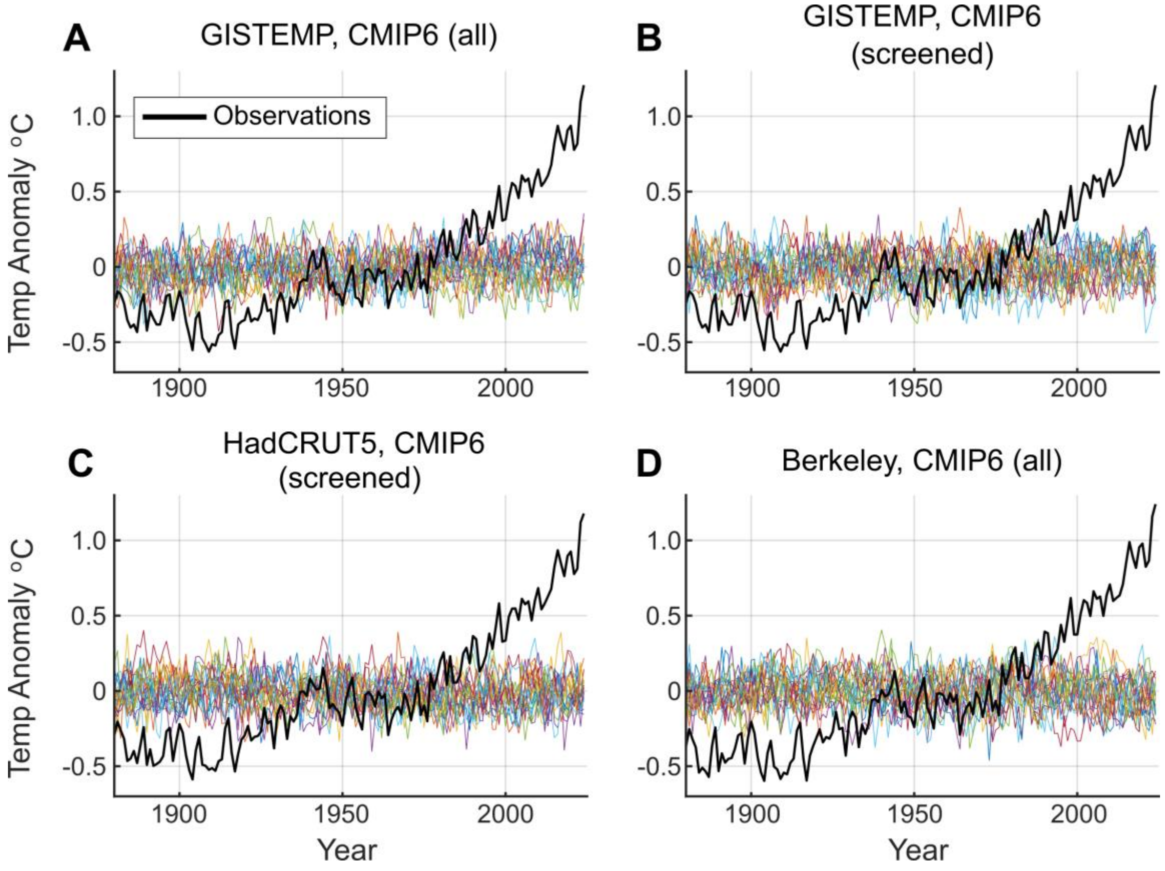

Fig. S7

As in Figure 3ab of main article, showing results for the other 4 experiments.

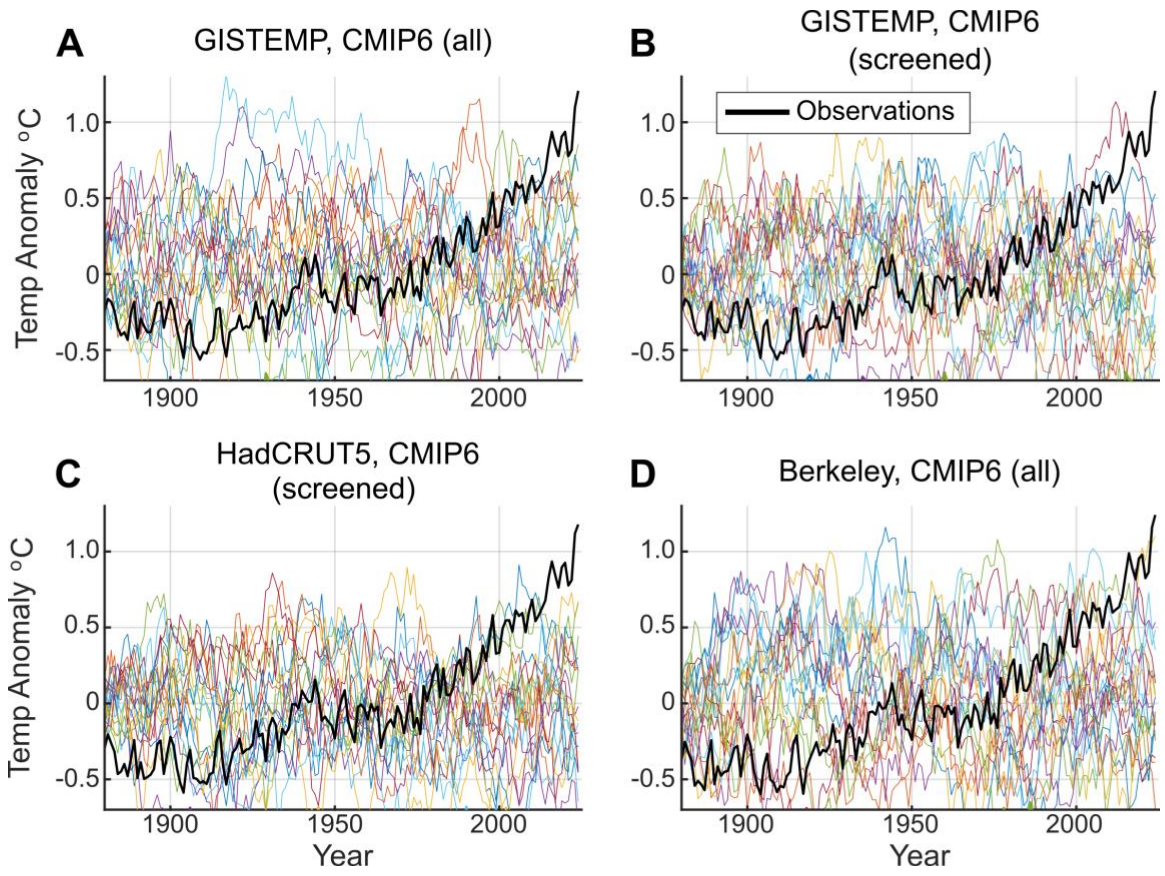

Fig. S8

As in Figure 3cd of main article, showing results for the other 4 experiments.

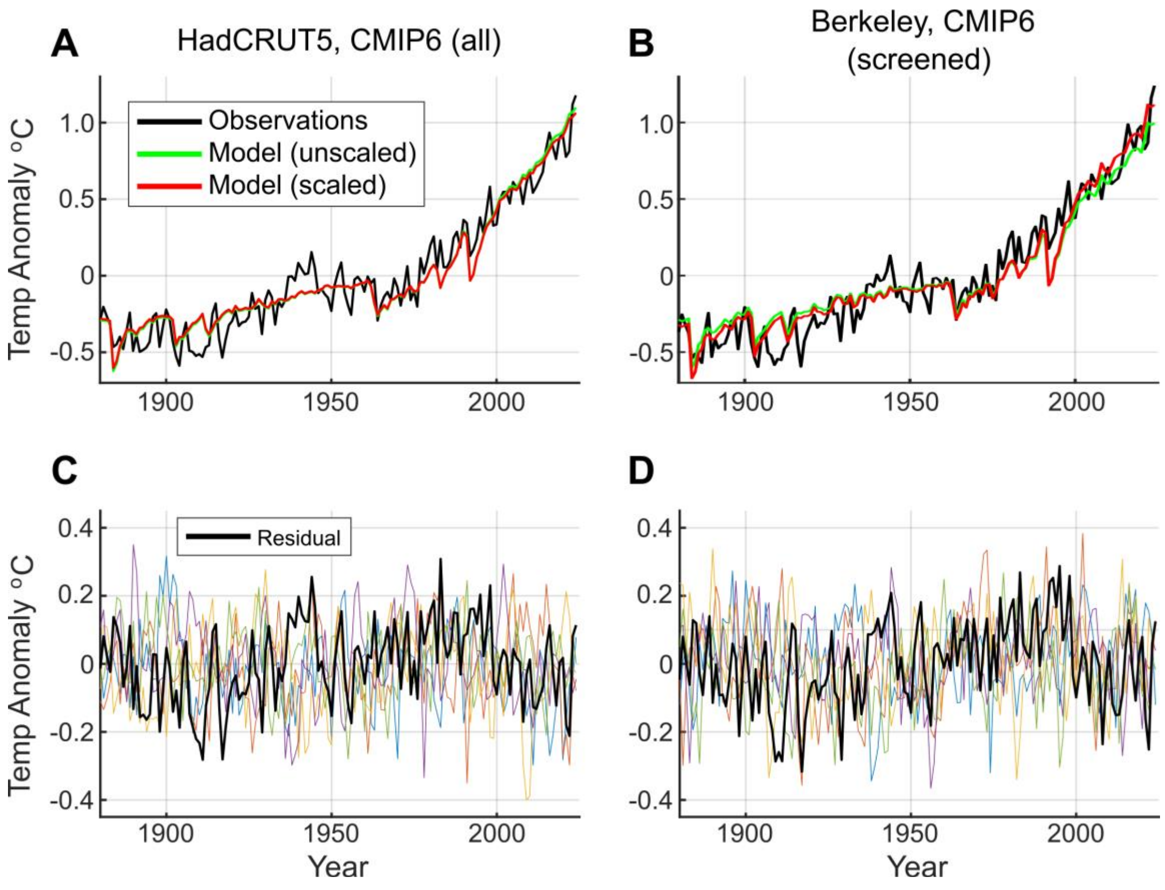

Fig. S9

As in Figure 1 of main article, but using BIC in place of AIC.

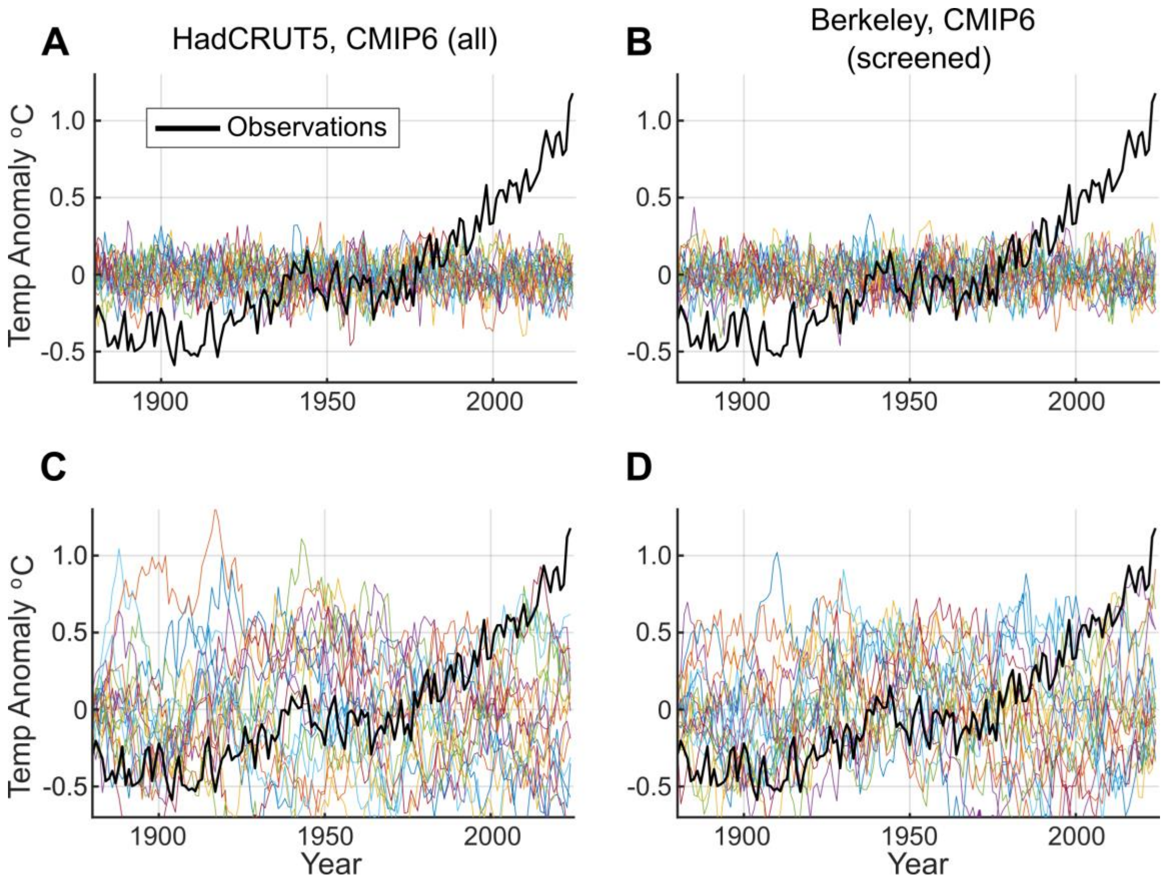

Fig. S10

As in Figure 2 of main article, but using BIC in place of AIC.

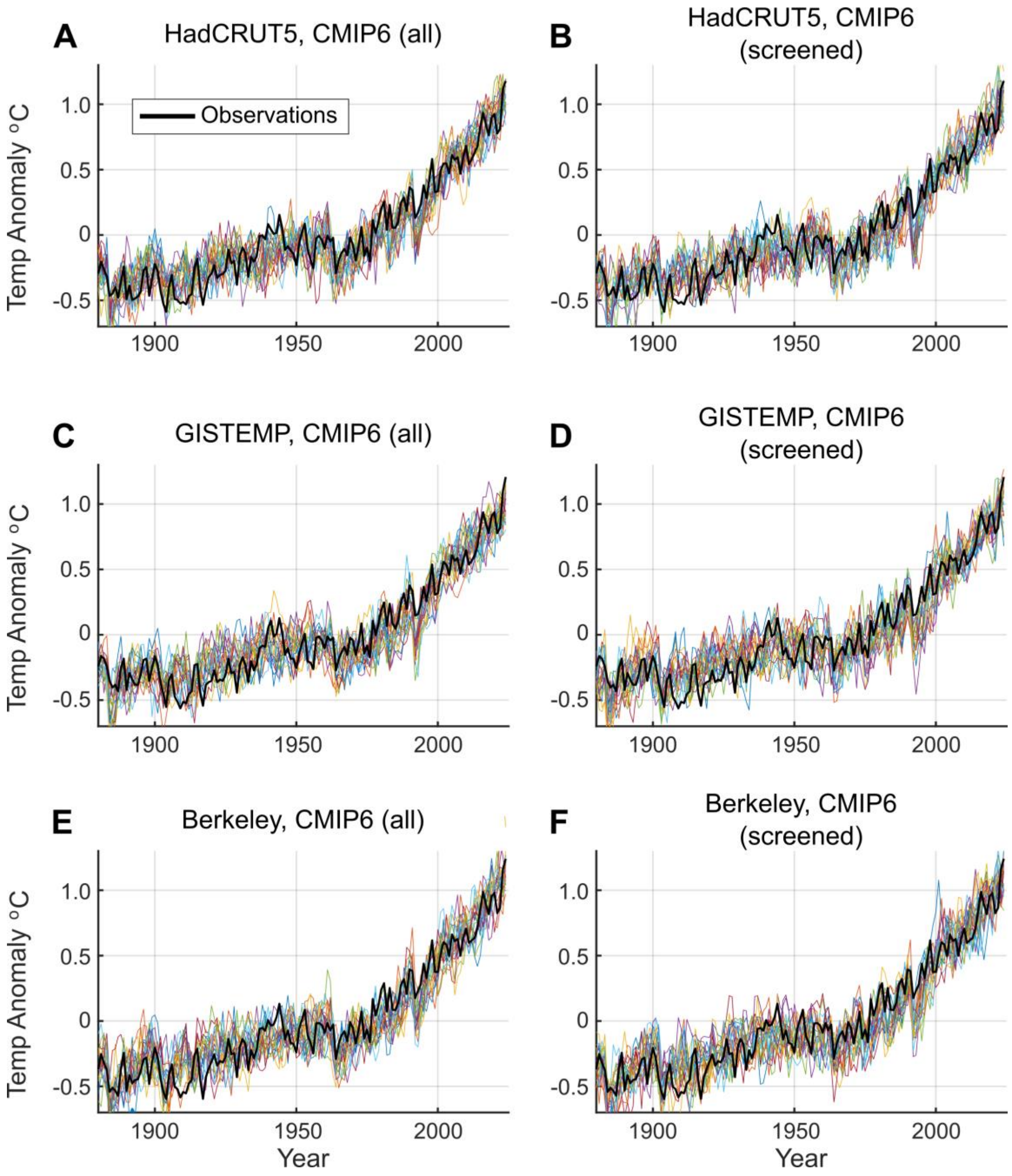

Fig. S11

As in Figure 3 of main article, but using BIC in place of AIC.

Table S1.
List of full set of CMIP6 Combined historical/SSP2-4.5 runs used in computing multimodel mean global temperature series used in this study. One (the first) realization is used for each indicated model. The “restricted” set (left) includes the $N = 33$ different models contained in the official LLNL CMIP6 multimodel archive while the “expanded” set (right) includes the $N = 44$ total models making additional use of the E.U. Copernicus CMIP6 multimodel archive The models with a “Y” in the TCR screen column are included in the screened subset of models.

Model	Source	TCR (°C)	TCR Screen (1.4-2.2°C)
**ACCESS-CM2**	**LLNL**	1.96	Y
**ACCESS-ESM1-5**	**LLNL**	1.97	Y
**AWI-CM-1-1-MR**	**LLNL**	2.03	Y
**BCC-CSM2-MR**	**LLNL**	1.55	Y
**CAMS-CSM1-0**	**LLNL**	1.73	Y
**CanESM5**	**LLNL**	2.71	N
**CanESM5-1**	**LLNL**	NA	N
**CAS-ESM2-0**	**LLNL**	NA	N
**CESM2-WACCM**	**LLNL**	1.91	Y
**CMCC-CM2-SR5**	**LLNL**	2.14	Y
**CMCC-ESM2**	**LLNL**	1.92	Y
**E3SM-1-1**	**LLNL**	2.9	N
**EC-Earth3**	**LLNL**	2.3	N
**EC-Earth3-CC**	**LLNL**	2.63	N
**EC-Earth3-Veg**	**LLNL**	2.66	N
**EC-Earth3-Veg-LR**	**LLNL**	NA	N
**FGOALS-f3-L**	**LLNL**	1.94	Y
**FGOALS-g3**	**LLNL**	1.5	Y
**FIO-ESM-2-0**	**LLNL**	2.22	N
**GFDL-ESM4**	**LLNL**	1.63	Y
**IITM-ESM**	**LLNL**	1.66	Y
**INM-CM4-8**	**LLNL**	1.3	N
**INM-CM5-0**	**LLNL**	1.41	Y
**IPSL-CM6A-LR**	**LLNL**	2.35	N
**KACE-1-0-G**	**LLNL**	2.04	Y
**MIROC6**	**LLNL**	1.55	Y
**MPI-ESM1-2-HR**	**LLNL**	1.64	Y
**MPI-ESM1-2-LR**	**LLNL**	1.82	Y
**MRI-ESM2-0**	**LLNL**	1.67	Y

**NESM3**	**LLNL**	2.72	N
**NorESM2-LM**	**LLNL**	1.49	Y
**NorESM2-MM**	**LLNL**	1.22	N
**TaiESM1**	**LLNL**	2.27	N
**CanESM5-CanOE**	**E.U. Copernicus**	2.71	N
**CESM2**	**E.U. Copernicus**	2	Y
**CIESM**	**E.U. Copernicus**	2.25	N
**CNRM-CM6-1**	**E.U. Copernicus**	2.22	N
**CNRM-CM6-1-HR**	**E.U. Copernicus**	2.46	N
**CNRM-ESM2-1**	**E.U. Copernicus**	1.83	Y
**HadGEM3-GC31-LL**	**E.U. Copernicus**	2.49	N
**KIOST-ESM**	**E.U. Copernicus**	NA	N
**MCM-UA-1-0**	**E.U. Copernicus**	1.9	Y
**MIROC-ES2L**	**E.U. Copernicus**	1.49	Y
**UKESM1-0-LL**	**E.U. Copernicus**	2.77	N

Table S2. Details of Experiments (CMIP6 Restricted/BIC) w/ Est Likelihoods (%) *

Experiment #	*ARMA(p,q)*	*β*	*1998*	*2005*	*2010*	*2014*	*2015*	*2016*	*2023*	*2024*
1. HadCRUT /CMIP6 All	AR(1)	0.97								
Anthropogenic + Natural			2.3	33	40	75	32	19	21	14
Natural (ARMA)			0	0	0	0	0	0	0	0
Natural (Persist Red Noise)			6.5	5.5	3.7	3.8	1.5	0.71	0.14	0.07
2. HadCRUT /CMIP6 Screen	AR(1)	1.05								
Anthropogenic + Natural			2.7	30	25	72	18	13	20	12
Natural (ARMA)			0	0	0	0	0	0	0	0
Natural (Persist Red Noise)			6.5	5.5	3.7	3.8	1.5	0.71	0.14	0.07
3. GISTEMP /CMIP6 All	AR(1)	0.94								
Anthropogenic + Natural			4.9	30	46	68	26	14	19	6.0
Natural (ARMA)			0	0	0	0	0	0	0	0
Natural (Persist Red Noise)			7.4	4.9	4.0	3.3	1.2	0.45	0.17	0.05
4. GISTEMP /CMIP6 Screen	AR(1)	1.02								
Anthropogenic + Natural			5.5	27	31	65	15	10	19	4.8
Natural (ARMA)			0	0	0	0	0	0	0	0
Natural (Persist Red Noise)			7.4	4.9	4.0	3.3	1.2	0.45	0.17	0.05
5. Berkeley /CMIP6 All	AR(1)	1.04								
Anthropogenic + Natural			2.4	30	49	78	41	20	30	18
Natural (ARMA)			0	0	0	0	0	0	0	0
Natural (Persist Red Noise)			6.4	5.3	4.3	4.2	1.8	0.75	0.24	0.11
6. Berkeley /CMIP6 Screen	AR(1)	1.12								
Anthropogenic + Natural			2.8	27	31	76	25	14	29	14
Natural (ARMA)			0	0	0	0	0	0	0	0
Natural (Persist Red Noise)			6.4	5.3	4.3	4.2	1.8	0.75	0.24	0.11

* “0” = No occurrences in 40,000 simulations

Table S3. Details of Experiments (CMIP6 Restricted/AIC) w Est Likelihoods (in %) *

Experiment #	*ARMA(p,q)*	*β*	*1998*	*2005*	*2010*	*2014*	*2015*	*2016*	*2023*	*2024*
1. HadCRUT /CMIP6 All	ARMA(1,2)	0.97								
Anthropogenic + Natural			2.2	31	38	73	30	17	19	13
Natural (ARMA)			0	0	0	0	0	0	0	0
Natural (Persist Red Noise)			6.5	5.5	3.7	3.8	1.5	0.71	0.14	0.07
2. HadCRUT /CMIP6 Screen	ARMA(1,2)	1.05								
Anthropogenic + Natural			2.3	29	23	70	17	12	18	10
Natural (ARMA)			0	0	0	0	0	0	0	0
Natural (Persist Red Noise)			6.5	5.5	3.7	3.8	1.5	0.71	0.14	0.07
3. GISTEMP /CMIP6 All	ARMA(1,2)	0.94								
Anthropogenic + Natural			4.1	28	44	65	24	12	18	5.5
Natural (ARMA)			0	0	0	0	0	0	0	0
Natural (Persist Red Noise)			7.4	4.9	4.0	3.3	1.2	0.45	0.17	0.05
4. GISTEMP /CMIP6 Screen	ARMA(1,2)	1.02								
Anthropogenic + Natural			4.7	25	28	62	12	8.`	16.4	4.1
Natural (ARMA)			0	0	0	0	0	0	0	0
Natural (Persist Red Noise)			7.4	4.9	4.0	3.3	1.2	0.45	0.17	0.05
5. Berkeley /CMIP6 All	ARMA(1,2)	1.04								
Anthropogenic + Natural			2.2	29	46	77	40	19	29	17
Natural (ARMA)			0	0	0	0	0	0	0	0
Natural (Persist Red Noise)			6.4	5.3	4.3	4.2	1.8	0.75	0.24	0.11
6. Berkeley /CMIP6 Screen	ARMA(1,2)	1.12								
Anthropogenic + Natural			2.6	27	30	75	24	14	28	14
Natural (ARMA)			0	0	0	0	0	0	0	0
Natural (Persist Red Noise)			6.4	5.3	4.3	4.2	1.8	0.75	0.24	0.11

* "0" = No occurrences in 40,000 simulations

Table S4. Details of Experiments (CMIP6 Expanded/BIC) with Est Likelihoods (in %) *

Experiment #	*ARMA(p,q)*	*β*	*1998*	*2005*	*2010*	*2014*	*2015*	*2016*	*2023*	*2024*
1. HadCRUT /CMIP6 All	AR(1)	0.97								
Anthropogenic + Natural			2.8	27	31	76	25	14	29	14
Natural (ARMA)			0	0	0	0	0	0	0	0
Natural (Persist Red Noise)			6.5	5.5	3.7	3.8	1.5	0.71	0.14	0.07
2. HadCRUT /CMIP6 Screen	AR(1)	1.05								
Anthropogenic + Natural			1.8	32	31	71	25	13	22	13
Natural (ARMA)			0	0	0	0	0	0	0	0
Natural (Persist Red Noise)			6.5	5.5	3.7	3.8	1.5	0.71	0.14	0.07
3. GISTEMP /CMIP6 All	AR(1)	0.94								
Anthropogenic + Natural			4.6	31	50	64	27	11	22	7.0
Natural (ARMA)			0	0	0	0	0	0	0	0
Natural (Persist Red Noise)			7.4	4.9	4.0	3.3	1.2	0.45	0.17	0.05
4. GISTEMP /CMIP6 Screen	AR(1)	1.02								
Anthropogenic + Natural			3.8	28	36	64	20	9	20	5.33
Natural (ARMA)			0	0	0	0	0	0	0	0
Natural (Persist Red Noise)			7.4	4.9	4.0	3.3	1.2	0.45	0.17	0.05
5. Berkeley /CMIP6 All	AR(1)	1.04								
Anthropogenic + Natural			2.3	32	52	75	43	18	33	20
Natural (ARMA)			0	0	0	0	0	0	0	0
Natural (Persist Red Noise)			6.4	5.3	4.3	4.2	1.8	0.75	0.24	0.11
6. Berkeley /CMIP6 Screen	AR(1)	1.12								
Anthropogenic + Natural			2.8	27	31	76	25	14	29	14
Natural (ARMA)			0	0	0	0	0	0	0	0
Natural (Persist Red Noise)			6.4	5.3	4.3	4.2	1.8	0.75	0.24	0.11
